# Evaluation of Mast Cell Density using CD117 antibody and Microvessel Density Using CD34 Antibody in Different Grades of Oral Squamous Cell Carcinoma

**DOI:** 10.31557/APJCP.2020.21.12.3533

**Published:** 2020-12

**Authors:** Fakeha Meraj Ansari, Muhammad Asif, Maryam Nazir Kiani, Nighat Ara, Muhammad Ishaque, Rabia Khan

**Affiliations:** 1 *Department of Histopathology, Armed Forces Institute of Pathology, Rawalpindi, Pakistan. *; 2 *Consultant Histopathologist, Armed Forces Institute of Pathology, Rawalpindi, Pakistan. *; 3 *Department of Oral Pathology, Army Medical College, Rawalpindi, Pakistan.*; 4 *Department of Orthodontics, Armed Forces Institute of Dentistry, Rawalpindi, Pakistan. *

**Keywords:** Mast cells, angiogenesis, oral squamous cell carcinoma

## Abstract

**Objective:**

To compare mast cell and microvessel densities among histologic grades of oral squamous cell carcinoma.

**Setting:**

Armed Forces Institute of Pathology. Materials and Methods: A total of 60 specimens of OSCC comprising 20 each of well, moderately and poorly differentiated were evaluated. Immunohistochemical analysis was performed to measure MCD and MVD by applying monoclonal CD117 antibody and monoclonal CD34 antibody, on formalin fixed and paraffin embedded sections. ANOVA and Post Hoc Tukey test was employed to assess the densities and to compare the differences between different grades of OSCC. A p-value <0.05 was considered to as significant.

**Results:**

There were 67% males and 33% females with a mean age of 60.1±16.0years. Immunohistochemical analysis revealed MCD to be 31.0±5.5 25.05±5.2, 10.90±3.5 in well, moderately and poorly differentiated OSCC. The intergroup comparison of decrease in MCD was also found to be statistically significant. The mean MVD was found to be 17.55±4.2, 20.35±3.6 and 28.60±3.2 in WDOSCC, MDOSCC and PDOSCC respectively. The pair wise result of MVD was found insignificant between well and moderately differentiated OSCC (p=0.057). However, the results of MVD was significant for well versus poorly differentiated and moderately versus poorly differentiated OSCC (p<0.001).

**Conclusion:**

The protective role of mast cells in OSCC is favored as a decrease in MCD is observed with the advancing histological grade of tumor. Significant results of MCD and MVD reveal that they can be used as an indicator for the disease progression in oral tumors This outcome might help delineating tumor population to get advantage from novel treatment modalities like mast cell degranulation blocking agents and anti-angiogenic therapy.

## Introduction

Oral Squamous cell carcinoma (OSCC) is one of the most common cancers known globally. In Pakistan, Shaukat Khanum Memorial Cancer Hospital, Lahore, also ranked oral cancer to be the third most common tumor reported (Badar and Mahmood, 2015). Despite the innovative advances made in the field of surgery, radiotherapy and chemotherapy, the survival rate of OSCC is still less than 50% (Marta Cristaldi et al., 2019). This is attributed to the late diagnosis of OSSC as reported by one of the studies claiming that almost half of the tumor cases are well advanced at the time of diagnosis concurrently leading to worse prognosis (Seoane-Romero et al., 2012). 

Tumor development and progression is a multistep and complex process. Angiogenesis is one of the main factor responsible for the initiation and progression of tumors. Tumors without their own microvasculature rarely grow more than few millimeters. Angiogenesis is triggered by an increase in angiogenic stimulators, for instance Vascular endothelial growth factor (VEGF), Basic Fibroblast growth factor (FGF), Tumor Necrosis factor (TNF) and transforming growth factor (TGF) or by the downpour of negative modulators, such as thrombospondin 1(TSP-1), angiostatin and endostatin. It is the result of an imbalance between these positive and negative factors that are produced by tumor and host cells.

Mast cells are the gate keepers of immune system and are one of the host cells responsible for angiogenesis. These highly versatile cells typically reside within the connective tissues and mucosal surfaces of the human body. Mast cells play a vital role in inflammation, tissue repair, angiogenesis and allergic reactions. The role of mast cells in tumor progression or its inhibition is still debatable. Its anti-tumor effects are linked to its main role and involvement in innate immunity. Nevertheless, some authors have proposed their role in progression of tumors principally due to their angiogenic ability. Mast cells are involved in the release of histamine as well as many angiogenic and pro-angiogenic factors. These include VEGF, β-FGF, TGF-β, and TNF-α. VEGF increases microvessels permeability and has pro-angiogenic effects. TNF-α is one of the most significant molecule causing tumor progression in the tumor vicinity released by MCs. Through the production of heparin which is an anticoagulant, Mast cells also modify hemostasis and blood perfusion in neoplasms (Soucek et al., 2007). Furthermore, the secretion of proteases by the MCs helps in degradation of extracellular matrix, formation of vascular tube, release of trapped angiogenic factors and promotion of metastasis. Mast cells play vital role by directly increasing mitotic activity of cancer cells as well as by indirectly altering microenvironment by aiding invasion. This activity is mainly mediated through heparin and TNF-α (Ashkavandi et al., 2010). Furthermore, chymase causes programmed cell death of different types of cells including endothelial cells that are mainly involved in the angiogenesis (Ozdemir, 2011). Another potential aid of mast cells in tumor promotion is by their mitogenic ability, deletion of tumor suppressor genes and stimulation of certain oncogenes via the c-kit locus (Nigrovic et al., 2008). Mast cells in addition to tumor growth have some anti-tumor effects as well which include restricting the cell growth, an amplified inflammatory response against tumors and through release of factors that decrease motility of tumor cells (Michailidou, Markopoulos and Antoniades, 2008).

In several studies, mast cells play a pro-tumorigenic role, whereas in others, they play an anti-tumorigenic role. Hence, the present study aims to find the densities of mast cells and micro vessels in different grades of oral squamous cell carcinoma.

## Materials and Methods

This comparative, cross sectional study was carried out in the Histopathology department, Armed Forces Institute of Pathology, Rawalpindi from June 2018 to December 2019. Overall, sixty cases of OSCC were retrieved along with their record files from the archives of Histopathology department. Twenty cases each of well, moderately and poorly differentiated OSCC were being evaluated. Approval of institutional review board was taken. Non-Probability convenience sampling was done. Poorly fixed, necrosed or autolysed tissue samples and tumors with scanty tissue were excluded from this study. The demographic details of patients were recovered from the history presented with each case. Paraffin embedded blocks were trimmed and cut into thin sections of 3-5 microns using microtome and mounted on slides. Hematoxylin and eosin (H&E) staining was performed on the tissue section slides. Tumor grades were identified and immunohistochemistry was carried out to determine the density of mast cells. For measurement of mast cell density (MCD) and microvessel density (MVD) tissue sections were reacted with monoclonal CD117 antibody (Leica, Microsystems, Germany) and monoclonal CD34 antibody (Leica, Microsystems, Germany) respectively as per the manufacturer’s guidelines. 

*Evaluation of CD117 Staining*

The initial qualitative evaluation was performed on all sections with Olympus BX53 microscope in order to examine the different expression pattern of the antibody. For quantitative analysis of MCD three microscopic high-power fields (HPFs) with highest vascularity (hotspots) were identified at low power (10x) whereas the mast cell count was done under an ocular grid at high power (40x). Images were captured with digital camera Olympus DP27. The hotspots which were closer to the invasive front of the tumor were preferred. All the mast cells immunoreactive to CD117 antibody were counted in selected hotspot field and the mean number of mast cells seen per HPF was recorded for particular case. Any cluster of mast cell granules appearing brown and clearly separated from adjacent cell membrane was considered to be a single mast cell. Results were verified by two observers to minimize bias. 

*Evaluation of CD34 Staining*

Microvessels were counted by the same counting method. Vessels were recognized by brown stained basement membrane around them. Partially identified vessels were not included in the count. 

*Statistical Analysis*

Results were analyzed using SPSS version 22.0. Descriptive statistics were used to describe the data. Mean and standard deviation was used to describe the numerical variables such as age, MCD and MVD. Categorical variables were analyzed by calculating frequency and percentages. ANOVA test was used to assess the significance of MCD and MVD among histological grades of OSCC. Post hoc Tukey test was applied for pair wise comparisons. A p-value of <0.05 was considered as significant.

## Results

A total of 60 cases 20 each of well, moderately and poorly differentiated were evaluated. There were 66.7% (n=40) males and 33.3% (n=20) female patients with a mean age of 60.10±16.01. Buccal mucosa was the most frequent tumor site accounting for 37.6% cases followed by tongue 

**Figure 1:**
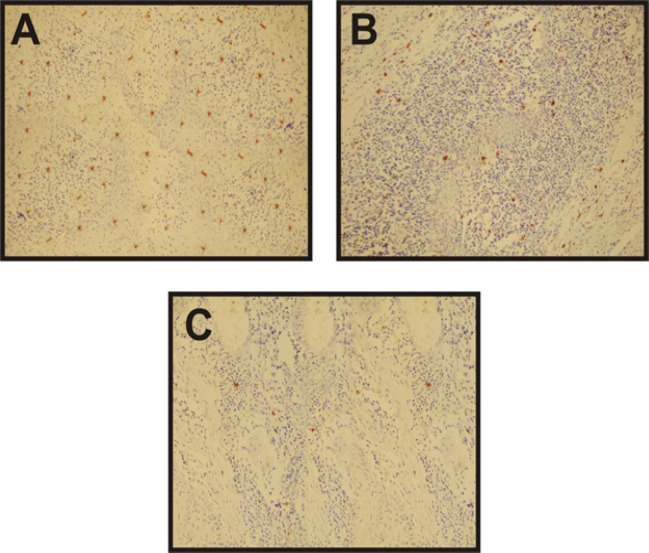
Photomicrograph Showing Expression of CD117 at 10x Magnification in Different Grades of OSCC. a) Well differentiated OSCC b) Moderately differentiated OSCC c) Poorly differentiated OSCC

On examination, all 60 cases of OSCC showed immunopositivity for CD117. The mean mast cell density was found to be 31.00±5.5, 25.05±5.2 and 10.90±3.5 in WDOSCC, MDOSCC and PDOSCC respectively. Mast cell density revealed a linear decrease as the grade of tumor advanced (Figure 1). One way ANOVA was applied on the results to compare the densities in different grades of OSCCs, p-value was found to be highly significant (p< 0.001) (Table 1).For pair wise comparison of results Post hoc Tukey test was applied and p-value was found to be significant in all the inter comparisons (p =0.001, p<0.001) (Table 2).

**Figure 2:**
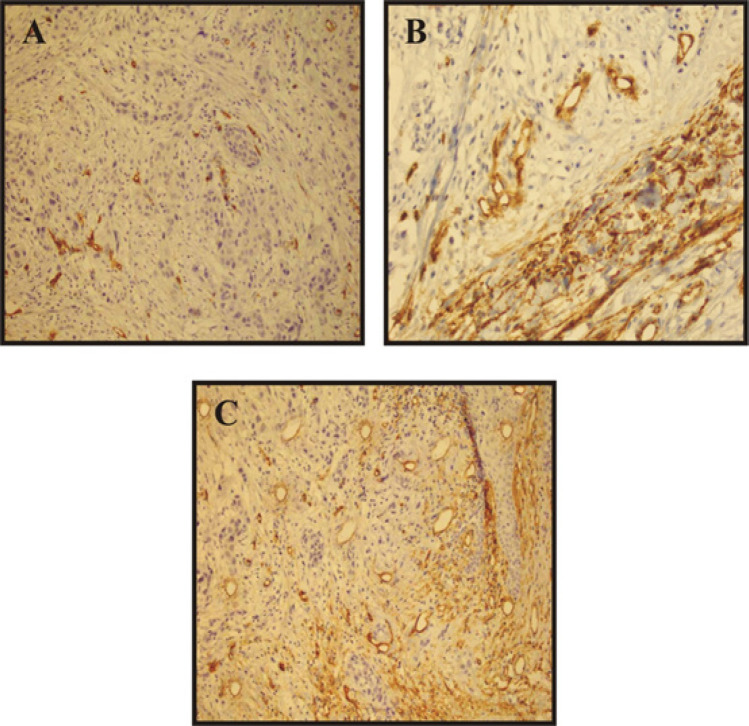
CD34 Showing Blood Vessels in Different Grades of OSCC. a) Well differentiated OSCC at 10x magnification b) Moderately differentiated OSCC at 40x magnification c) Poorly differentiated OSCC at 10x magnification

The mean MVD was found to be 17.55±4.2, 20.35±3.6 and 28.60±3.2 in WDOSCC, MDOSCC and PDOSCC respectively. MVD revealed a linear increase as the grade of tumor advanced (Figure 2). One-way ANOVA was applied on the results to compare the densities in different grades of OSCCs, p-value was found to be highly significant (p< 0.001) (Table 3). For pair wise comparison of results Post hoc Tukey test was applied (Table 4). It was observed that there was no significant difference between well and moderately differentiated OSCC (p=0.057). However, the results were significant for well versus poorly differentiated and moderately versus poorly differentiated OSCC (p<0.001). 

**Figure 3:**
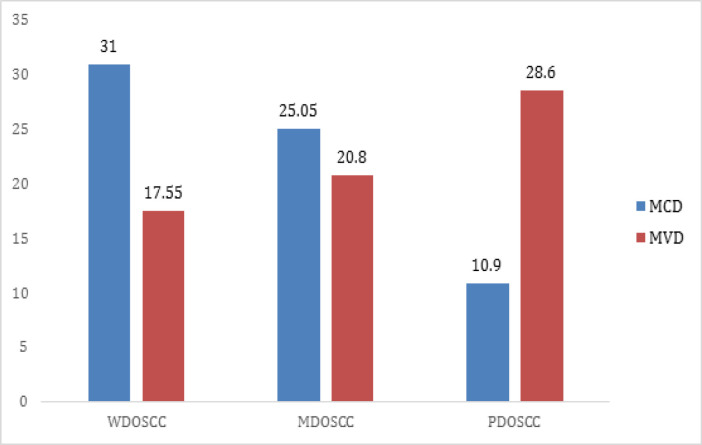
Bar Chart Showing Relation of MCD and MVD in Different Grades OSCC. MCD mast cell density; MVD microvessel density

A decrease in MCD was observed as the grade of tumor advanced. However, MVD increased with the advancement of tumor grade (Figure 3).

**Table 1:** Mean Mast Cell Density in Different Grades of OSCC

MCD	n	Mean	Standard deviation	F	p-value
Well differentiated	20	31.0	5.55	96.856	<0.001
Moderately differentiated	20	25.05	5.26		
Poorly differentiated	20	10.90	3.58		

**Table 2:** The Comparison of MCD in Different Grades of OSCC

Grade	Difference in count	P value	Confidence Interval
Well vs. moderately	5.376	0.001	2.45-8.30
Well vs. poorly	18.7	<0.001	16.53-23.67
Moderately vs. poorly	13.324	<0.001	10.58-17.72

**Table 3:** Mean Microvessel Density in Different Grades of OSCC

MVD	n	Mean	Standard deviation	F	p-value
Well differentiated	20	17.55	4.21	33.76	<0.001
Moderately differentiated	20	20.8	3.64	33.76	<0.001
Poorly differentiated	20	28.6	3.21	33.76	<0.001

**Table 4:** Comparison of MVD in Different Groups of OSCC

Grade	Difference in count	p-value	Confidence Interval
Well vs. moderately	2.8	0.057	0.08 to -6.58
Well vs. poorly	11	<0.001	7.72 to 14.38
Moderately vs. poorly	8.25	<0.001	4.47 to 11.13

## Discussion

For the tumor progression, an imbalance occurs between the positive and the negative angiogenic factors which are produced both by the tumor cells as well as the host cells resulting in angiogenesis. Mast cells are among the host cells that produce and release angiogenic factors. 

Microvessel density can be observed in wide array of tumors by the use of several endothelial markers. The most common utilized markers being VEGF, CD105, CD31, CD34 and von Willebrand factor (VWF) (Ashkavandi et al., 2010). CD34 is a glycosylated cell surface glycoprotein that is selectively expressed on hematopoietic progenitor and endothelial cells and has the potential to show small, large, and newly formed vessels. 

Mast cells act as an independent prognostic factor in various tumors such as those of prostate, melanoma, pancreatic cancer, and leukemia leading to poor prognosis (Johansson et al., 2010; Ribatti et al., 2003; Strouch et al., 2010; Molica et al., 2003). In 2008, a study was being carried out on the tumors of breast which revealed the presence of stem cell factor and c-kit factor. These two factors play a crucial role for the survival and growth of MCs (Rajput et al., 2008). However, in contrast, few studies suggest a good prognosis and better survival chances with the increased number of mast cells in certain soft tissue sarcomas, colorectal cancer and pulmonary adenocarcinoma (Dundar et al., 2008) (Väyrynen et al., 2013).

A study carried out in Norway described that a low MCD predicts poor prognosis and less survival chances of OSCC patients and suggested that MCD could be used as an easy and practical tool for prognostic evaluation (Attramadal et al., 2016). 

In 2014, Zaidi and colleagues assessed the role of mast cells in OSCC and they found out that these cells may be targeted in a new treatment regimen for the tumors through the selective inhibition of angiogenesis, tissue remodeling and tumor promoting molecules. Mast cells serve as a novel therapeutic target for cancer treatment and that inhibiting mast cell function may also inhibit tumor growth (Zaidi and Mallick, 2014). 

In another study influence of Tryptase-positive (MCT+) and CD117-positive (CD117+) mast cells on clinicopathological parameters and on the survival of OSCC patients was evaluated. They observed that competing risk regression showed a noteworthy influence of the resection status (R) on the incidence of first local recurrence while high MCD in tumor associated stroma indicated a long overall survival (Brockmeyer et al., 2017).

Since the role of mast cells in tumor stroma is very controversial so dissimilar results have been reported in different studies. In another study MCD was compared among OSCC, premalignant oral lesions as well as their relationship with clinical and microscopic parameters. No correlation could be found out between MCD with clinical and microscopic characteristics of OSCC. They concluded that migration failure of particular cells due to modification in the microenvironment in the course of tumor advancement could be the reason behind the decline in the number of mast cells. (Oliveira-Neto et al., 2007) Iamaroon et al., (2003) carried out a comparison of the MCD in normal oral mucosa as well as in different grades of dysplasia and OSCC. An increase in the densities of microvessels and mast cells with the progression in the stage of disease was observed in their study. They assumed mast cells to be the probable cause by increasing angiogenesis in OSCC. However, in case mast cells were solely responsible for angiogenesis, they would have also increased exponentially as the grade of tumor advanced rather than being decreased, circuitously suggesting part of other mediators regulating angiogenesis as well.

Ribatti (2016) proposed that mast cells can be new therapeutic targets in neoplasms because they release cytotoxic cytokines leading to the inhibition of neovascularization and tissue remodeling in tumors.

To date, there has been a great development in the field of chemotherapeutic agents to combat the challenge of cancer treatment but many of these drugs have limited efficacy. The main reason being the problem of their delivery, penetration and relatively less selectivity for the tumor cells which causes great damage to healthy tissues. Moreover, the heterogeneity of the tumor cells along with their genetic instability and high rate of mutation makes them a challenging target. As a result, drug resistant tumor cells proliferate. Another approach to address the same issue was reported by Kalra et al., (2012). This approach, described as “anti-angiogenic therapy,” aims at targeting activated endothelial cells. Since the selection of a suitable target is a prerequisite for designing any therapeutic agent, the advantage of exploiting these cells as drug target is twofold. One, the target must be genetically stable, diploid and homogenous, and secondly it should not be prone to spontaneous mutation. Another prerequisite of this therapy is that the target should be certainly reachable by systemic administration as the therapy is focused towards the activated endothelial cells.

Multi-institutional studies with larger sample size are recommended to be carried out in future to determine the relationship of MCD and MVD with survival and prognosis in OSCC and developing various adjuvants therapeutic strategies like anti-angiogenesis or mast cell degranulation blockage therapy. 

Hence, we conclude that significantly increased densities of the mast cells in well and moderately differentiated OSCC as compared to poorly differentiated OSCC strongly advocate that they may be used as an indicator of disease progression in oral tumors. Moreover, the protective role of mast cells in OSCC is favored as a decrease in MCD is seen with the advancing histological grade of tumor. This outcome might help delineating tumor population, which might get advantage from novel treatment modalities like the mast cell degranulation blockage agents. Furthermore, increased MVD in poorly differentiated OSCC as compared to well and moderately differentiated OSCC suggests that it can be used as an additional criterion to histologically grade the tumors and also provide objective assessment for therapeutic strategies.
